# Hemodynamic Effects of Combined Focal Cerebral Ischemia and Amyloid Protein Toxicity in a Rat Model: A Functional CT Study

**DOI:** 10.1371/journal.pone.0100575

**Published:** 2014-06-27

**Authors:** Jun Yang, Christopher D. d'Esterre, Zareen Amtul, David F. Cechetto, Ting Yim Lee

**Affiliations:** 1 Imaging Laboratories, Robarts Research Institute, Department of Medical Biophysics, University of Western Ontario, London, Ontario, Canada; 2 Department of Radiology, Foothills Medical Center, University of Calgary, Calgary, Alberta, Canada; 3 Department of Anatomy and Cell Biology, University of Western Ontario, London, Ontario, Canada; 4 Imaging Division, Lawson Health Research Institute, London, Ontario, Canada; University of Münster, Germany

## Abstract

**Background/Objective:**

Clinical evidence indicates that cerebral ischemia (CI) and a pathological factor of Alzheimer's disease, the β-amyloid (Aβ) protein, can increase the rate of cognitive impairment in the ageing population. Using the CT Perfusion (CTP) functional imaging, we sought to investigate the interaction between CI and the Aβ protein on cerebral hemodynamics.

**Methods:**

A previously established rat model of CI and Aβ was used for the CTP study. Iodinated contrast was given intravenously, while serial CT images of sixteen axial slices were acquired. Cerebral blood flow (CBF) and blood volume (CBV) parametric maps were co-registered to a rat brain atlas and regions of interest were drawn on the maps. Microvascular alteration was investigated with histopathology.

**Results:**

CTP results revealed that ipsilateral striatum of Aβ+CI and CI groups showed significantly lower CBF and CBV than control at the acute phase. Striatal CBF and CBV increased significantly at week 1 in the CI and Aβ+CI groups, but not in the Aβ alone or control group. Histopathology showed that average density of dilated microvessels in the ipsilateral striatum in CI and Aβ+CI groups was significantly higher than control at week 1, indicating this could be associated with hyperperfusion and hypervolemia observed from CTP results.

**Conclusion:**

These results demonstrate that CTP can quantitatively measure the hemodynamic disturbance on CBF and CBV functional maps in a rat model of CI interacting with Aβ.

## Introduction

Stroke and Alzheimer's disease (AD) are the most common contributors to cognitive impairment in the population greater than 65 years of age [1]. The pathogenic mechanisms of these two conditions not only overlap, but are also highly interactive [2]. In fact, the presence of ischemic lesions or silent infarcts in persons with AD is associated with a greater decline in cognition [3]–[5]. It is speculated that cerebral ischemia (CI) may accelerate AD disease progression [3]–[8].

For patients with moderate to severe stages of AD and vascular dementia, cerebral hypoperfusion is prevalent [9]–[12]. Changes in cerebral blood flow (CBF) occur early in the pre-symptomatic stages of AD, much sooner than brain atrophy, tau and plaque pathology [10], [13]–[15]. Some subjects with mild cognitive impairment (MCI) also show a similar pattern of hypoperfusion, in the absence of substantial Aβ plaque [10], [16]. Animal studies have shown that CI can stimulate mRNA expression of amyloid precursor protein (APP) and APP proteolytic processing to β-amyloid protein (Aβ), a central neuro-toxic/degenerative factor in AD pathogenesis [17], [18]. Disruption of the blood-brain-barrier (BBB) caused by CI may also increase the extravasation of soluble Aβ peptides, as well as its precursor APP, into the brain parenchyma, resulting in a neuroinflammatory reaction and Aβ plaque formation [19], [20]. In turn, Aβ accumulation can reduce brain capillary density and cause aberration of capillary structures, decreasing local cerebral perfusion [21]–[25]. These hemodynamic changes may indicate neurovascular degeneration [26].

CT perfusion (CTP), a functional imaging modality involving intravenous injection of contrast agent, is currently used for the diagnosis of both acute stroke and brain tumors [27], [28]. CTP not only measures tissue perfusion but also vascular permeability, an indicator of BBB integrity. Moreover, this technique is more accessible and less expensive to perform in the clinic than single photon emission computed tomography (SPECT) and positron emission tomography (PET), the modalities currently used for studying dementia. CTP-derived CBF and cerebral blood volume (CBV) can clearly reveal the degree and site of ischemia in a relatively short scanning time with minimal invasiveness.

We sought to determine the potential negative hemodynamic effects of Aβ toxicity combined with CI. To mimic the clinical situation, an intra-cerebroventricular injection of Aβ_25–35_ fragment, and unilateral striatal ischemic insult were conducted in an animal model [29], [30]. CTP imaging was performed to visualize and measure CBF and CBV, in conjunction with histology. We hypothesize that the combination of Aβ toxicity and CI will cause greater hemodynamic disruption compared to CI alone or control.

## Methods and Materials

### Animals

Male Wistar rats, weighing 250–300 g, were obtained from Charles River (Montreal, Quebec). They were housed in separated cages in a room maintained at 23°C with light from 7:00 to 19:00 hr, and had free access to food and water. All experimental procedures were approved by the Animal Use Subcommittee of the Canadian Council on Animal Care of our institution (protocol number: 2008-113). At end of the study, all animals were euthanized by administration of pentobarbital overdose (80 mg/kg) and perfused transaortically, first with 0.01M PBS and then followed by 4% paraformaldehyde (pH 7.4). The brains were carefully removed and cryoprotected in 30% sucrose at 4°C before sectioning.

### Surgical procedure

Rats were anesthetized with 2–2.5% isoflurane/medical air during surgery. A stereotaxic frame was used for all surgeries and body temperature was maintained at 37°C. The atlas of Paxinos and Watson was used for selecting the stereotactic coordinates for all injections. Small burr holes were drilled in the parietal bone at near-bregma locations to insert injection cannula (23-gauge). Rats were divided into 4 groups: (1) CI model: unilateral injection of 60 pmol vasoconstrictor, endothelin-1 (ET) (Sigma-Aldrich, Oakville, ON) into right striatum (anterior/posterior (AP): +0.5 mm relative to bregma, medial/lateral (ML): −3.0 mm relative to bregma, and dorsal/ventral (DV): −5.0 mm below dura); (2) Aβ+CI model: first bilateral injections of 50 nmol Aβ_25–35_ peptide (Bachem, Torrance, CA) into lateral ventricles (AP: −0.8 mm relative to bregma, ML: ±1.4 mm relative to bregma, and DV: −4.0 mm below dura) followed by the same unilateral ET injection into right striatum; (3) Amyloid alone model (Aβ alone): bilateral injections of 50 nmol Aβ_25–35_ peptide into lateral ventricles was used as an internal control for comparison between Aβ+CI and Aβ alone model; (4) Sham-control: unilateral injection of 10 µL of 0.9% w/v saline into right striatum as in the CI model. At the end of each injection, the cannula was left in-situ for 3 minutes before fully retracted. Once all the injections were completed, the wound was sutured and each rat received one dose of intramuscular buprenorphine (40 mg/kg).

### CT perfusion scanning

CTP studies were performed at pre-surgery baseline, 30 min, 60 min, 1 week and 4 weeks post-surgery on rats which were anesthetized with 1.5% isoflurane during the scans. Each CTP study started with an injection of iodinated contrast agent (Isovue-300, Bracco Diagnostics, Princeton, NJ) at a dose of 2.5 mL/kg body weight into a tail vein at an infusion rate of 8 mL/min while a clinical CT scanner (GE Healthcare, Waukesha, WI) continuously scanned coronal sections of the rat brain using the high resolution mode. The technical parameters used were FOV of 10 cm, 80 kVp, 300 mA and 0.4 s per rotation of the gantry. Each CTP acquisition consisted of two phases: 24 scans acquired every 1 second, and 12 scans acquired every 14.6 seconds. Sixteen image slices (1.25 mm thick/slice) were scanned for each study. CBF and CBV maps were generated with the CT Perfusion software (GE Healthcare, Waukesha, WI) [31].

### Image post-processing and analysis

The average maps of all acquired images of each CTP study were manually co-registered to a digital 3D atlas template of the rat brain (LONI rat brain atlas, UCLA, CA) by alignment of corpus callosum, lateral ventricles and cerebellum using Analyze v11.0 software (Mayo Clinic, Overland Park, KS). Region of interests (ROIs) were defined in the striata of the same coronal slices of the brains from all experimental groups. Absolute values of CBF and CBV were obtained from the defined striatal ROIs. ROI data from each time point were then normalized in two ways: 1) either with its contralateral value for the groups with unilateral injection of ET and control to differentiate effects of Aβ+CI and CI from control, or 2) with its pre-surgery baseline value for the comparison between Aβ+CI and Aβ alone group.

### Immunohistochemistry

Immunohistochemical staining was performed on serial, coronal sections of the entire brain and 35 µm-thick sections were cut using a Tissue-Tek Cryo3 sliding microtome (Torrance, CA). Sections were then stained with laminin primary antibody (1∶1000, rabbit anti-rat Laminin, Sigma-Aldrich). Laminin staining was used to measure numbers and diameters of microvessels. The stained brain sections were then examined using a light microscope (Leica DC-300, Heerbrugg, Switzerland). The results were expressed as the numbers of dilated microvessels per mm^2^ of the striatum.

### Statistical analysis

Normalized CBF and CBV between baseline and other time points were analyzed by using one-way ANOVA and Tukey's post hoc tests with a significance level of *p*<0.05. A two-group comparison of the hemodynamics between Aβ+CI and Aβ alone group for each time point, was assessed by t-test with *p*<0.05. All histological measurements were analyzed by using one-way ANOVA followed by Dunnett's post hoc tests with *p*<0.05. All values were presented as mean ± standard error of the mean (SEM).

## Results

### CTP functional maps

**Figure 1:**
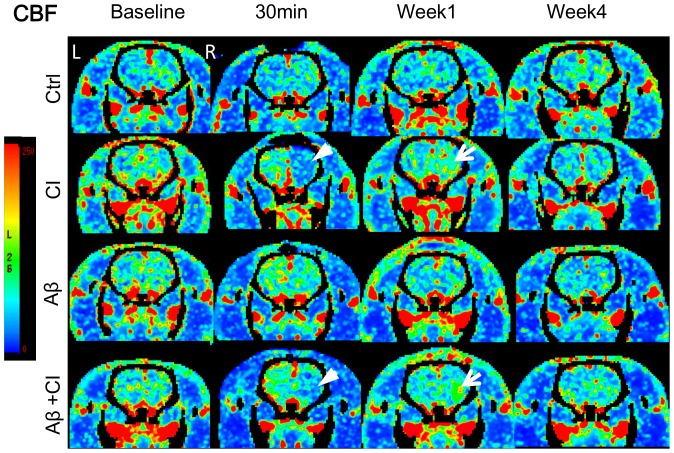
Cerebral blood flow maps at four time points in: control rat (1^st^ row); CI rat (2^nd^ row); Aβ alone rat (3^rd^ row) and Aβ+CI rat (4^th^ row). Baseline imaging was done before any injection. In CI and Aβ+CI brains, ischemia (white arrow head) and hyperperfusion (white arrow) in the right striatum were observed at 30 minutes and 1 week post injection, respectively. No significant change in CBF was observed over four weeks in control and Aβ alone rats, when compared to their baselines.

**Figure 2:**
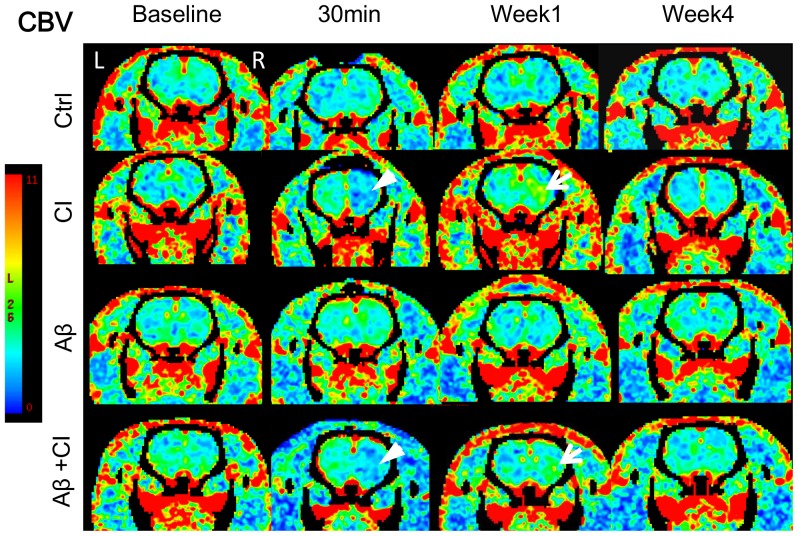
Cerebral blood volume maps at four time points in: control, CI, Aβ alone and Aβ+CI rat. Baseline imaging was done before any injection. In CI and Aβ+CI brains, similar to the CBF results, CBV deficit (white arrow head) and hypervolemia (white arrow) in the right striatum were observed at 30 minutes and 1 week post injection, respectively. Similar to the CBF maps, no significant CBV change was observed over four weeks in control and Aβ alone rats, when compared to their baselines.

CTP-derived CBF and CBV maps at baseline, 30 min, week 1 and week 4 post injection of one rat from each of the four groups are shown in figure 1 and 2, respectively. Baseline CBF and CBV among all groups were not significantly different. In the CI group there was a large ischemic lesion at 30 min post injection, which showed as large CBF and CBV defects in the functional maps. The Aβ+CI brain also showed a large hypoperfused lesion at 30 min, mainly in the right striatum. Increased CBF (hyperperfusion) and CBV (hypervolemia) were observed at week 1 in both CI and Aβ+CI animals, but not in control. The animal with Aβ alone injection did not show significant changes of CBF and CBV from baseline over 4 weeks. No significant difference in CBF and CBV at week 4 was observed between CI and Aβ+CI group.

### Cerebral ischemia and hyperperfusion post ischemia

**Figure 3:**
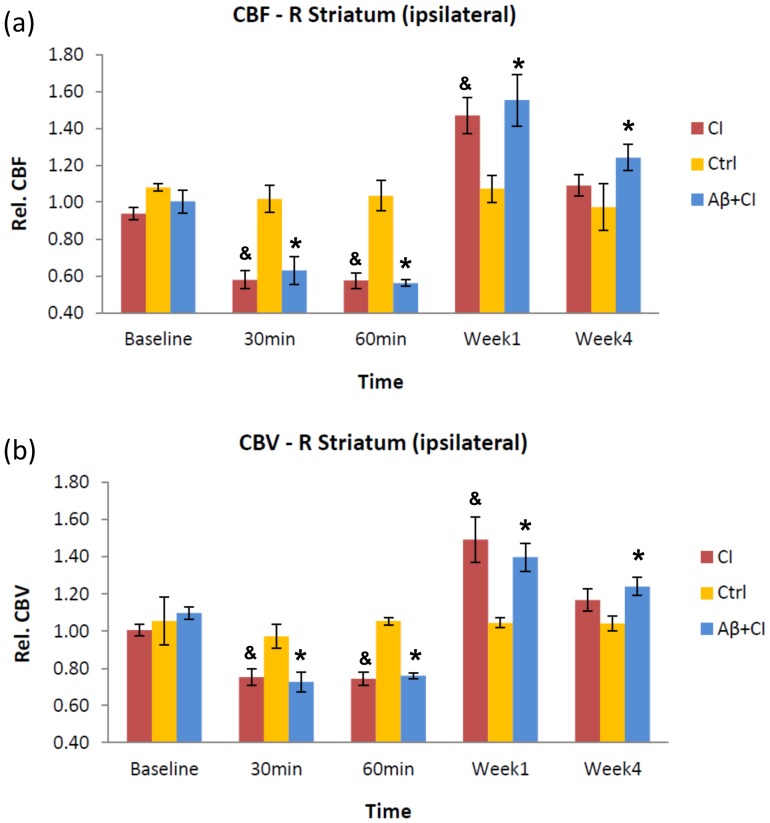
Evolution of striatal CBF and CBV over four-week period post injection. Absolute CBF and CBV in the right (ipsilateral) striatum at each time point from Aβ+CI, CI and control group were normalized by their contralateral values. (a), normalized (relative) CBF; (b), normalized CBV. In Aβ+CI group (*, n = 7), there were significant differences in CBF and CBV between baseline and those at other time points (30 min, 60 min, week 1 and week 4. *p*<0.05). Similar findings were shown in the CI group (&, n = 6. *p*<0.05), except for week 4. No significant CBF and CBV difference from baseline was found in the control group (n = 3). In addition, Aβ+CI and CI groups showed significantly lower CBF and CBV at acute phase and higher CBF and CBV at week 1 than control. However, no significant difference between Aβ+CI and CI group was seen over 4 weeks.

Relative CBF (rCBF) and CBV (rCBV) to the contralateral striatum in the control group (n = 3) did not show significant differences between baseline and other time points (Fig. 3a and b). In contrast, Aβ+CI (n = 7) and CI (n = 6) groups at the acute phase (30–60 minutes) had a significantly lower rCBF and rCBV in the right striatum when compared to their baseline values as well as to control (*p*<0.05). At week 1, rCBF and rCBV increased significantly from baselines in the right striatum of the CI (*p*<0.05) and Aβ+CI (*p*<0.05) groups, but not in the control group. Furthermore, at week 4 only the combined Aβ+CI group showed a significantly higher rCBF and rCBV in the right striatum when compared to its baseline (*p*<0.05). However, no significant difference between Aβ+CI and CI group was seen over 4 weeks.

### Comparison of hemodynamics between Aβ+CI and Aβ alone model

**Figure 4:**
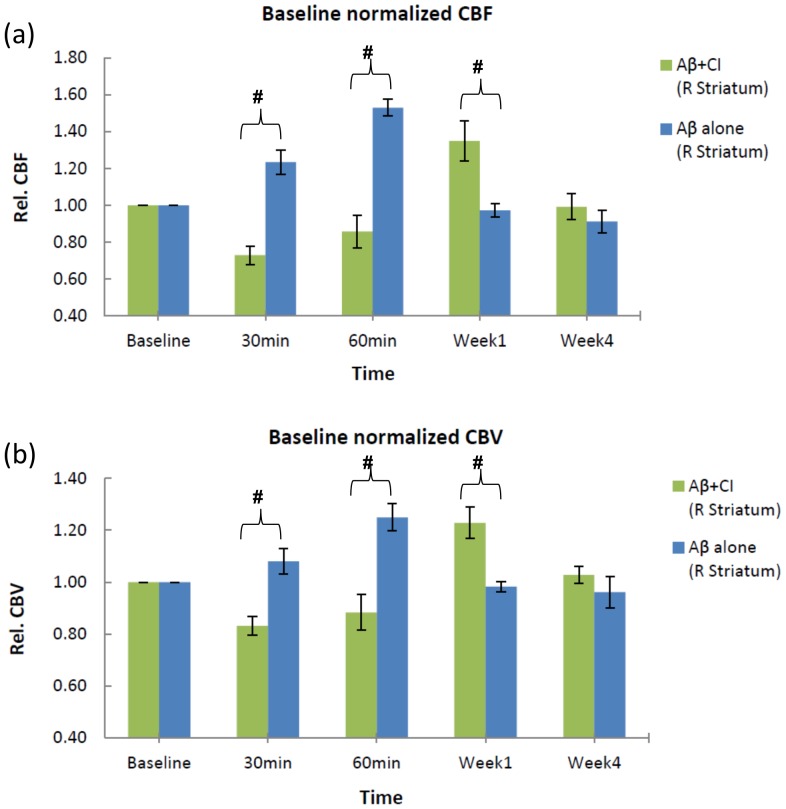
Hemodynamic effects of Aβ+CI and Aβ alone models. For rats which had combined Aβ and ET injections (n = 7) and Aβ only injections (n = 6), absolute CBF and CBV in the right (ipsilateral) striatum were normalized with their respective baseline values. Both relative CBF (a) and CBV (b) were significantly different between the Aβ+CI and Aβ alone group at the time points post insult except for week 4 (#, *p*<0.01 for 30–60 min, *p*<0.05 for week1). At week 4, rCBF and rCBV in both Aβ+CI and Aβ alone groups dropped to the baseline level.

To differentiate hemodynamic effects caused by combined Aβ and CI to that by Aβ alone, ipsilateral (right striatum) CBF and CBV normalized by their baseline (pre-surgery) values between Aβ+CI and Aβ alone group were compared (Fig. 4a and b). At 30–60 minutes and 1 week, but not 4 weeks post the insult, ipsilateral rCBF and rCBV in the striatal ROIs from Aβ+CI model (n = 7) were significantly different from those from Aβ alone model (n = 6). Aβ+CI group showed an opposite temporal changes in ipsilateral rCBF and rCBV to the Aβ group at the acute state (*p*<0.01) and week 1 (*p*<0.05). At the first week, hyperperfusion and hypervolemia were seen in the Aβ+CI group, but not in the Aβ group. At week 4 the hyperperfusion and hypervolemia in the Aβ+CI group had subsided to be statistically non-significant from its baseline. Interestingly, from week 1 to week 4, ipsilateral rCBF and rCBV of Aβ+CI group decreased much faster than those of Aβ alone group (−36±−11% versus −6±−9% for CBF; −20±−7% versus −2±−6% for CBV).

### Vascular pathology after induced CI and Aβ

**Figure 5:**
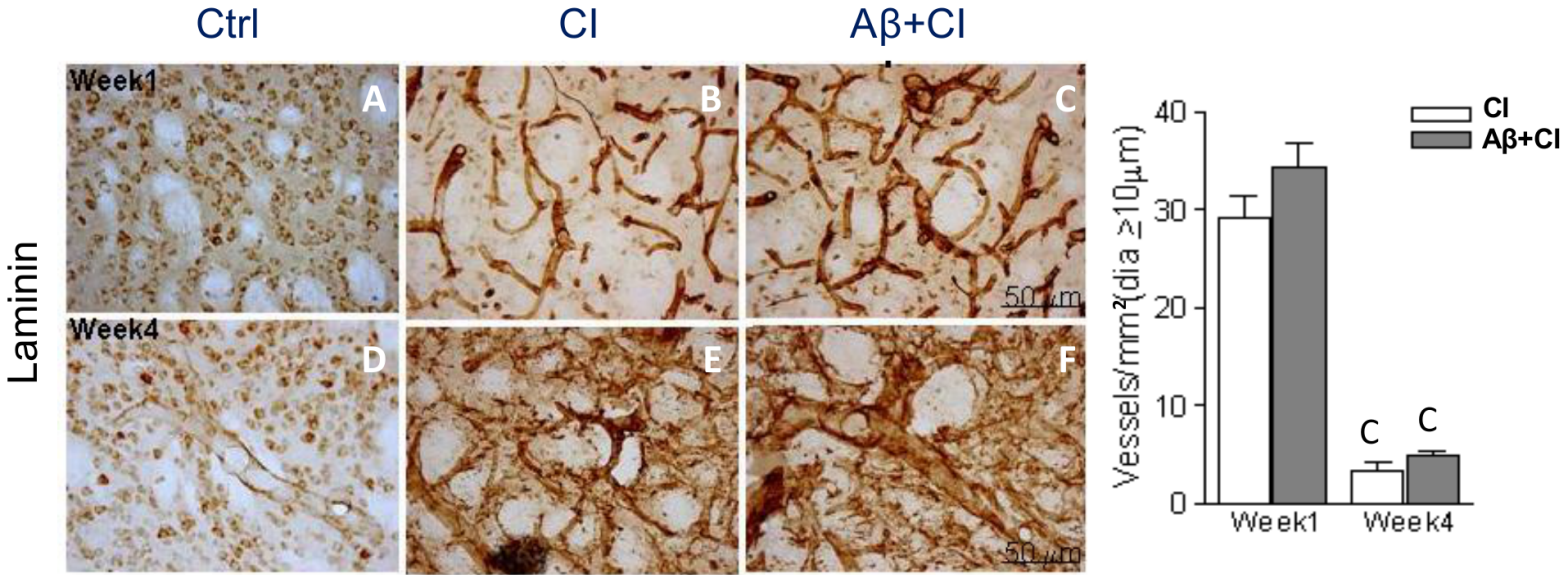
Histology of cerebral microvessels in control, CI and Aβ+CI groups. Microphotographs showed laminin-stained microvessels in the core of ipsilateral (right) striatum at week 1 (A–C) and week 4 (D–F) post insults. Quantitative analysis of the whole striatal core showed that average density of dilated microvessels (i.e. diameter greater than 10 µm) in CI and Aβ+CI groups was significantly higher at week 1 than those at week 4. As induced injury advanced, at week 4 regular vasculature was almost absent in the Aβ+CI group. Letter “C” indicates significant differences when compared to the control group, *p*<0.05.

Viable microvessels assessed by laminin staining was determined in the right (ipsilateral) striatum of control (n = 3), CI (n = 6) and Aβ+CI (n = 6) groups. At week 1, a diffused network of laminin-positive vessels as a result of leakage was detected in the core lesion of CI and Aβ+CI brains. The presence of dilated microvessels (with a diameter greater than 10 µm) was also observed surrounding the lesion epicenter at the striatum in CI and Aβ+CI groups (Fig. 5, B and C). However, at week 4, laminin immunoreactivity was observed extensively around damaged microvessels in the lesion of CI and Aβ+CI brains (Fig. 5, E and F), and these brains also had a reduction in dilated vessels. The average number of dilated microvessels per mm^2^ in the core of right striatum was 29±2 for CI and 34±3 for Aβ+CI group at week 1, but this number significantly decreased to 3±1 and 5±1 for CI and Aβ+CI, respectively at week 4.

## Discussion

The results herein support the hypothesis that the addition of ischemic insult to Aβ pathology leads to greater hemodynamic dysfunction. The major findings are as follows: first, CTP imaging had successfully showed a significant decrease of CBF and CBV in the ipsilateral striatum at the acute phase, followed by a post-ischemic hyperperfusion and hypervolemia at week 1 in Aβ+CI and CI groups. Second, for the two-group comparison between Aβ+CI and Aβ alone model, the ipsilateral striatum affected by Aβ+CI had significant differences in both CBF and CBV compared to that of Aβ alone model from acute phase to week 1. Aβ alone group did not show the hyperperfusion and hypervolemia at week 1, in contrast to the Aβ+CI group. Third, laminin staining showed increased vasodilation at week 1 as a result of reperfusion reaction, which was related to the hyperperfusion in Aβ+CI and CI groups.

Previous work on neurodegeneration has focused on structural alternations, such as brain atrophy and cerebroventricular enlargement using routine CT and MRI [32], [33]. However, this diagnostic approach is limited by the low sensitivity and specificity to detect early functional changes [33]. Conversely, a reduction in glucose metabolism, detected by functional imaging using ^18^F-FDG PET, has been shown to occur years before onset of clinical symptoms [34]–[36]. Prior to the hypometabolic state, Aβ accumulation in the brain is hypothesized to be the primary driving factor in AD-related pathogenesis [37]. Several PET studies have shown that the levels of ^11^C-PIB (i.e. radiotracer which binds to Aβ aggregates) retention can be used to differentiate between patients with AD and/or mild cognitive impairment (MCI) and healthy individuals [33], [36], [38]. However, the ability of Aβ imaging to diagnose early AD rests upon the assumption that Aβ plays a central role in the progression of the disease. Some subjects exhibit typical Aβ pathology without clinical symptoms, similarly 25–35% of healthy individuals over the age of 75 years show cortical ^11^C-PIB uptake [33], [38], [39], suggesting that Aβ is not the only crucial driving factor for cognitive impairment. Agreed with this view, variably sized cerebrovascular defects are frequently present with AD-related pathogenesis and cognitive decline [4], [32], [40]. Recent studies show that the evolution of changes in cerebral perfusion does not necessarily corroborate with gross structural changes of the neurodegenerative brain [9], [23]. Brain hypoperfusion and endothelial dysfunction likely precede the hypometabolic and neurodegenerative state observed in MCI and AD [9]–[11], [14], [16]. As such, CTP imaging may be used to characterize these changes of causative cerebral hemodynamics.

A hyper-acute decrease, followed by an increase in CBF and CBV in the brains of the Aβ+CI group may reflect a dynamic transition from normal cognition/perfusion to a compensatory brain mechanism which attempts to revive the impaired neurovascular functionality prior to substantial neurodegeneration and amyloid deposition. For both Aβ+CI and CI groups, CBF and CBV parameters showed a similar decreasing trend within 60 minutes after the injection, indicative of successful CI induction by ET. Hyperperfusion and hypervolemia were present in the right striatum at week 1 for both Aβ+CI and CI groups, possibly due to release of vasodilators elicited by CI. There were no significant inter-group differences found between the Aβ+CI and CI group at the acute state and week 1–4, indicating that at this phase of disease progression, CI acted as a dominant driving factor in causing hemodynamic dysfunction. This result is supported by a previous investigation which indicates that the development of pathological changes, changes in infarct size, or even cognitive deficits do not fully develop until 3 weeks after the insult [30]. With more prolonged interaction between Aβ pathology and CI, only the Aβ+CI group demonstrated significant intra-group CBF and CBV differences between week 4 and its baseline level, a trend not observed in the control and CI groups.

The presence of soluble Aβ proteins could further increase cellular stress and reduce vascular tone via the inflammatory cascade when CI coexists [29], [30]. Focal CI has also been shown to produce larger infarcts in transgenic mice overexpressing APP [41], [42]. Aβ-induced vascular dysregulation, which may increase the propensity for ischemic damage, threatens overall cerebral perfusion [43]. This is partially consistent with our findings in which reactive hypervolemia appears at the first week as a post-ischemic reperfusion response in the Aβ+CI model. Recent clinical studies using ASL or PW-MRI also revealed similar states of hyperperfusion in the hippocampus, cingulate gyrus, amygdala and striatum of patients with MCI and mild AD [44]–[46]. Our histopathology data showed that an increase in microvessel diameter, distributed sporadically throughout the striatal ischemic core at week 1, is consistent with vasodilation to maintain regional cerebral perfusion in response to the drop in CBF and CBV after ischemia was induced in CI and Aβ+CI rats.

We also compared longitudinal hemodynamics of ipsilateral striatum between Aβ+CI and Aβ alone group. For the Aβ alone group, the initial CBF and CBV increase at the acute state and later decrease at week 1 and week 4 may be attributed to an immediate response to the injection, and later followed by a vasoconstriction induced by this soluble Aβ [25], [47], [48], respectively. However, the moderate amount (50 nmol) of Aβ used in this experiment might not be the optimal dose to maintain the vasoconstrictive effect for four weeks or longer duration than expected. In contrast, the Aβ+CI group showed the opposite hemodynamic effect. This may suggest that hyperperfusion and hypervolemia after CI were a result of prominent hemodynamic disturbance which was further amplified by the initiation of an Aβ-induced pro-inflammatory response. From week 1 to 4, CBF and CBV within Aβ+CI group declined much faster than those of Aβ-only group, indicating that adding Aβ could greatly attenuate the reactive hypervolemia triggered by CI.

Two main limitations of the study included: first, the size of the rat brain relative to the resolution of the clinical CT scanner might contribute to the variability involved in the map processing and registration. However, in our study a high resolution mode was used during CTP scans and this may help to compensate for that limitation. A small animal phantom scanned under the same mode showed an achievable spatial resolution of 500 µm (data not shown), which should be sufficient in guiding ROI placement in a large anatomic region such as striatum in the rat brain here. The feasibility of CTP in evaluating CI has been validated against PET. CTP-derived CBF measurements have shown a good correlation with PET-derived CBF values [49]–[51]. In addition, CTP imaging can be combined with vasodilatory challenge using acetazolamide to assess cerebrovascular reserve in acute stroke, which may further help to identify penumbra and infarct core [52]. In the other hand, dynamic susceptibility contrast MRI (DSC-MRI) or MR perfusion has also been applied in assessment of cerebrovascular reserve using acetazolamide. Although MR perfusion has similar or even higher spatial resolution and larger coverage of the brain than CTP, changes in MR signal intensity are not linearly related to changes in contrast concentration, resulting in difficulty with measurement of absolute perfusion parameters in detecting perfusion defect [52], [53]. For the second limitation, as vascular cognitive impairment is an insidious disease process, a study is needed to elucidate the long-term effect of CI on Aβ. Moreover, the addition of contemporaneous PET-CTP imaging is needed.

In summary, we showed that the co-existence of CI and Aβ disrupted normal cerebral perfusion and exacerbated post-ischemic injury, when compared to the control or Aβ alone. The observed hyperperfusion and hypervolemia post CI support the assertion that there is a local compensatory brain mechanism which occurs early in the pathological progression. This compensation is further associated with increased amount of dilated microvessels. The subsequent decrease in CBF and CBV reflects the failure of vascular autoregulation after Aβ and CI initiated the inflammatory cascade. Overall, this study demonstrated that CTP-derived CBF and CBV are suitable parameters for quantitatively assessing variable hemodynamic changes in the early stage of cerebral ischemia when neurotoxic Aβ is present.
